# PEGylated Protamine Letrozole Nanoparticles: A Promising Strategy to Combat Human Breast Cancer via MCF-7 Cell Lines

**DOI:** 10.1155/2022/4438518

**Published:** 2022-06-10

**Authors:** Muhammad Tafhim Khan, Zia Uddin, Muhammad Arslan Javed, Nabi Shah, Hamid Bashir, Ahson Jabbar Shaikh, Muhammad Shahid Riaz Rajoka, Muhammad Imran Amirzada, Muhammad Hassham Hassan Bin Asad

**Affiliations:** ^1^Department of Pharmacy, COMSATS University Islamabad, Abbottabad Campus, 22060 Abbottabad, Pakistan; ^2^Department of Medicine, Services Institute of Medical Sciences, Services Hospital, Lahore, Pakistan; ^3^Centre for Applied Molecular Biology, University of the Punjab, Lahore, Pakistan; ^4^Department of Chemistry, COMSATS University Islamabad, Abbottabad Campus, 22060 Abbottabad, Pakistan; ^5^Food and Feed Immunology Group, Laboratory of Animal Food Function, Graduate School of Agricultural Science, Tohoku University, Sendai 980-8572, Japan; ^6^Institute of Fundamental Medicine, Department of Genetics, Kazan Federal University, Kazan, Russia; ^7^Institute of Neurophysiopathology (INP), Aix-Marseille Universite, Marseille, France

## Abstract

The objective of the study was to develop PEGylated protamine letrozole nanoparticles to combat human breast cancer by modifying the release pattern of letrozole. Breast cancer is amongst the most prevalent diseases in women due to overactivity of human epidermal growth factor receptor 2 (HER2). PEG-protamine letrozole nanoparticle formulation was designed and optimized to alter the release pattern of the drug. The size, morphology, and structure of PEG-protamine letrozole NP were characterized by FTIR, XRD, Zetasizer, and SEM analysis. The result showed the PEG-protamine letrozole nanoparticles were irregular in shape and have size ranging from 258 nm to 388 nm, polydispersity index 0.114 to 0.45, zeta potential of 11.2 mV, and entrapment efficiency 89.93%. XRD studies have confirmed that the crystal structure of letrozole has become amorphous. The drug release study maintained the prolonged release for 72 hours. Moreover, the PEG-protamine letrozole NPs displayed a strong anticancer action compared to MCF-7 cells with an IC50 70 *μ*M for letrozole and 50 *μ*M for PEG-protamine letrozole NPs. Overall, our results indicate that letrozole PEG-protamine NPs alter the release profile of letrozole, which could be an excellent approach for overcoming letrozole resistance in human breast cancer.

## 1. Introduction

Breast cancer is globally known as the most common prevalent disease in women and classified as overfunctioning of human epidermal growth factor receptor (HER2). Overexpression of HER2 protein is associated with tumor aggression, prognosis, and reactivity to hormonal and cytotoxic agents in breast cancer patients [1]. HER-2 and estrogen receptors (ER) are routinely examined in breast cancer because of an indication of crossover talk between ER and HER-2 pathway. ER*α* interact with HER-2 protein, leading to the initiation of Mitogen-activated Protein Kinase (MAPK) signaling pathway [2, 3]. Aromatase inhibitors (AIs) are exploited effectively in the therapy of ER*α* active breast cancer by reducing the interconversion of estrogens and androgens [4]. It is very unfortunate that resistance to AIs is still very common in individuals with metastatic tumors [5]. Letrozole, which is used as an aromatase inhibitor, lowers the concentration of estrogen by inhibiting estrogen production. Letrozole upregulates ER*α* and induces activation of MAPK through inhibition of estrogen synthesis [6]. Several strategies have been adopted to treat cancer and combat its fatal complications. Green synthesis and biosynthesis of silver nanoparticles using an extract of oak fruit hull (Jaft) and extract of olive leaf reported higher activity on MCF-7 [7, 8]. Incorporation of a protein with PEG would facilitate to escape the immune system recognition and hinder the in vivo clearance of plasma [9]. Nanoparticles, PEGylated with targeting ligands, are the most recent methodology for targeted drug delivery. The success of protein PEGylation is a valuable strategy to enhance bioavailability and more efficacious treatment of a targeted disease [10, 11]. Recently, PEG has been used in many drug delivery systems (DDS) as well as has been used as site-selective carrier which has the ability to deliver proteins, peptides, and genes. The aim of the current research work was to design PEG-protamine letrozole nanoparticles to modify the release profile of letrozole and improve the anticancer effect.

## 2. Materials and Methods

### 2.1. Materials

Letrozole was donated by Rotex Pharmaceutical Industry, Islamabad, Pakistan. Sodium tripolyphosphate protamine sulphate, N-hydroxysuccinimide, and 1-ethyl-3-(3-dimethylaminopropyl) carbodiimide hydrochloride (EDC-HCL) were purchased from Sigma.

### 2.2. PEG-Protamine Complex Preparation

Protamine and PEG (0.1%) solution was formulated using purified water. NHS and EDC-HCL solutions were blended with the solution of PEG, and solution of protamine was dropwise added to the PEG solution. The subsequent mixture was stirred at a speed of 800 rpm for at least 5 hours. Later, that mixture was added to the dialysis membrane of molecular cutoff value 1000 Dalton and left for 72 h for dialysis. The dialyzed solution was lyophilized, and freeze-dried powder of PEG-protamine complex obtained.

### 2.3. Preparation of Letrozole (LTZ) Loaded PEG-Protamine Nanoparticles

PEG-protamine complex (0.3%) solution was prepared. Letrozole 05 mg, 10 mg, and 15 mg were dissolved in ethanol. Sodium tripolyphosphate (TPP) 0.4% solution was prepared and added slowly and drop by drop with continuous shaking at a speed of 500 rpm leading to formation of PEG-protamine complex. Letrozole was then added dropwise at baseline temperature into this mixture. The solution was then shaken for at least 5 hours at a speed of 800 rpm and centrifuged at 12000 rpm at a temperature of 15°C. Settled particles were then gathered while removing the supernatants.

### 2.4. PEG-Protamine Nanoparticle Characterization

#### 2.4.1. FTIR Analysis of PEG-Protamine Complex and Loaded Nanoparticles

The PEG-protamine complex was characterized by FTIR, in which the pallets were prepared by KBr (1 : 08) using a pellet press. The spectrum was collected and interpreted accordingly (FTIR-8400 Shimadzu). Similarly, PEG-carboxylate, letrozole, PEG-protamine complex, protamine sulphate, blank nanoparticles, and drug-loaded PEG-protamine letrozole NPs were also characterized by FTIR (Nicolet 6700™, Thermo Fisher Scientific); spectra were logged in the wavelength scale of 4500-400 cm^−1^ [12].

#### 2.4.2. Particle Size, Zeta Potential, and Polydispersity Index

The nanoparticle size and zeta potential of the formulations were determined using Malvern Zetasizer ZS (Malvern Instrument, Worcestershire, United Kingdom). The average diameter and polydispersity index (PDI) of nanoparticles in a homogenized mixture were defined by using the dynamic light scattering (DLS) technique [13, 14]. A sample of 10 *μ*l/ml was prepared for particle size evaluation. Dried out nanoparticles were distributed in deionized water and were sonicated for a minimum of 5 min for the proper blending of mixture [15].

#### 2.4.3. X-Ray Diffraction (XRD) Analysis

Purity and crystal-like properties of nanoparticle powder were assessed with the help of SmartLab SE XRD (Rigaku Asia Pacific Pet LTD, Singapore). The sample was filled in the sample holder, glass slide was exploited for the smoothing surface, while, for the scanning purpose, copper was utilized (ranging from 10 to 70) with diffractive meter and peaks were noted [16].

#### 2.4.4. Scanning Electron Microscopy (SEM)

The nanoparticle surface properties were examined by SEM. The nanoparticles were lyophilized and spread over the aluminum slice, which was covered with gold about 300 Å thick. Surface morphology and properties were reported for the sample analyzed at 10 kV [17].

### 2.5. Letrozole Measurement

The quantity of letrozole in the formulation was determined indirectly. The LTZ quantity from the supernatants was based on the absorbance of sample 240 nm. A standard curve was drawn, and absorbance of the sample was measured in 5 ml quartz cuvettes using Shimadzu UV-1700 Pharma spec UV-visible spectroscopy [18]. Samples obtained at various intervals were analyzed by UV-visible spectroscopy at 240 nm [19].

### 2.6. Drug Release Study

Drug release form nanoparticles was conducted by utilizing dissolution apparatus II. Sample amounts of 5 ml were withdrawn from the dissolution apparatus and replaced by fresh buffer solution at intervals of 5 min, 10 min, 20 min, 40 min, 1 h, 2 h, 4 h, 6 h, and 8 h. Various models including Higuchi, Korsmeyer-Peppas, and zero-order and first-order kinetics were utilized for dissolution data analysis. Pharmacokinetic characteristics of the drug can be concluded generally utilizing the zero-order kinetics mathematically represented as *Q*_0_–*Q*_*t*=_ *K*_0_*t*, where *K*_0_ stands for the rate constant, *Q*_*t*_ is the amount of drug released at time *t*, and *Q*_0_ is the initial amount of drug in nanoparticles. First-order kinetics can be expressed mathematically in *DQ*/*dt* = −*K*_1_*Q*, where*K*_1_represents the first-order kinetic rate constant and *Q* = log*Q*_0_–*k*_1_*t*/2.303. The Higuchi model sets out the drug release process as a dependent process based on Fickian diffusion from the polymer matrix. The Korsmeyer-Peppas model describes the administration of the drug from polymeric substances obtained from a simple relationship, according to the value of “*n*” which determines the release of the drug from the polymeric matrix [18].

### 2.7. Yield (% *w*/*w*)

Weight by weight (*w*/*w*) percentage was concluded from the weight of the lyophilized dried powder acquired from all batches. The retrieved nanoparticles were weighted and then divided by sum of the initial materials [20].

(1) $$\begin{array}{c} Y\text{ield }\left(\%\right)=\frac{\text{weight of dried nanoparticles recovered}}{\text{PEG}‐\text{protamine}+\text{cross}‐\text{linker}+\text{LTZ}}. \end{array}$$

### 2.8. Cytotoxicity Assay

#### 2.8.1. Preparation of Samples

The dried nanoparticles were dispersed in 100% DMSO (dimethyl sulfoxide) in an amount of 20 mg/ml; working solution of all the samples was prepared in DMEM at a concentration of 2 mg/ml which was further serial diluted.

#### 2.8.2. Antiproliferative Assay

The nanoparticles were tested against MCF-7 (human breast adenocarcinoma cells) for their cytotoxic activity by utilizing dimethyl–2–thiazolyl–2,5–diphenyl–2*H*–tetrazolium bromide- (MTT-) based cell sustainability assay. Cells were cultured for 48 hours to achieve the desired densities of the cells and finally treated with different concentrations of letrozole and PEG-protamine letrozole NPs (10-140 nM). Control cells were treated with 0.1% dimethyl sulfoxide (DMSO). The plates were kept for 24 h in an incubator, set for cell culture. Media was removed, and cells were treated with 20 *μ*g/mile end concentration of MTT prepared in the media. The plate was kept in a CO_2_ incubator at 37°C for 4 h, and then, 100 *μ*l DMSO was added to solubilize formazan crystals. Absorbance reading was taken by using a microplate reader (BMG, FLUOstar Omega, Germany) at 570 nm and 630 nm. Background reading (630 nm) was subtracted from 570 nm reading. The whole experiment was performed in triplicate, and the results are calculated as percent inhibition following the formula%cytotoxicity = 100 − [absorbance of sample/absorbance of untreated control × 100).

## 3. Results and Discussion

### 3.1. FTIR Analysis of PEG-Protamine Complex

**Figure 1:**
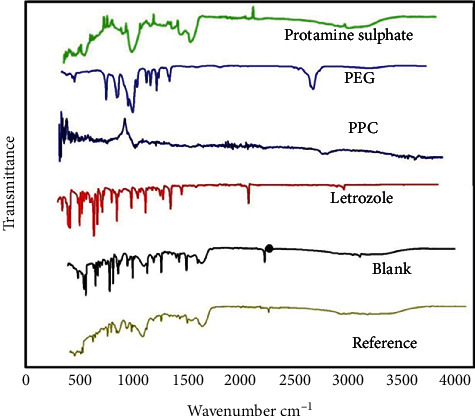
FTIR spectra of protamine, polyethylene glycol (PEG), PEG-protamine complex (PPC), letrozole, blank nanoparticles, and letrozole-loaded nanoparticles.

Characterization of the PEG-protamine complex and nanoparticles was effectuated with FTIR spectral analysis, which confirmed the formation of PEG-protamine complex; this analysis resulted in several characteristic peaks at 1522.76 cm^−1^, 1623.44 cm^−1^, and 2320.73 cm^−1^ representing the typical peaks of protamine (Figure 1), whereas the PEG-carboxylate represents characteristic crests at 1278.7 cm^−1^, 1339.50 cm^−1^, and 1466.01 cm^−1^ displayed in Figure 1. As shown in the figure, a novel peak at 1633.36 cm^−1^ validates the chemical mismatch between protamine sulphate and PEG-carboxylates that makes PEG-protamine compound. The occurrence of corresponding crests at 1633 and 1278 cm^−1^ validated the creation of an amide bond in an amine functional group of protein and carbonyl functional group of PEG carboxylate. LTZ was then loaded into the PEG-protamine complex utilizing cross-linked TPP. Alterations observed in the peaks of the PEG-protamine complex and LTZ interaction between PPC and LTZ [13].

Encapsulation of letrozole in the PEG-protamine complex was also confirmed by FTIR analysis. As shown in Figure 1, principal crests at 2232.6 cm^−1^ for C≡N stretching, 3054.27 cm^−1^ for sp2 hybridized CH extending, and 690-900 cm^−1^ for out-of-plane CH distortion modes of vibration pointed out the functional groups. The PEG-protamine complex shows characteristic peaks at 1466.20 cm^−1^, 1663.36 cm^−1^, and 2882.44 cm^−1^; similarly, the blank PEG-protamine nanoparticles displayed characteristic peaks at 1200.34 cm^−1^, 1600.45 cm^−1^, and 2334.12 cm^−1^. Moreover, the FTIR of drug-loaded nanoparticles as shown in Figure 1 displayed characteristic peaks at 1300.45 cm^−1^, 2245.34 cm^−1^, and 2930.45 cm^−1^ subsequently, on drug loading to PEG-protamine nanoparticles which resulted in a new peak at 2190.75 cm^−1^ that highlighted the mismatch in PEG-protamine complex and LTZ [16].

### 3.2. X-Ray Diffraction Analysis (XRD)

**Figure 2:**
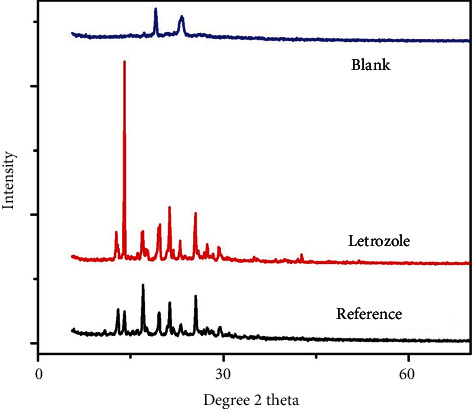
XRD analysis for letrozole, blank nanoparticles, and drug-loaded PPC.

**Figure 3:**
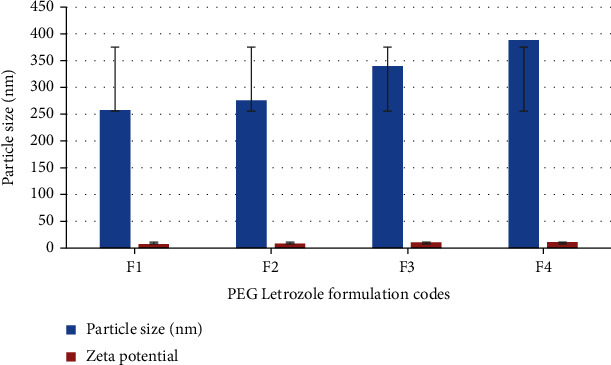
Particle size and zeta potential of PGE-letrozole formulations.

To further characterize and explore other physicochemical properties, X-ray diffraction analysis of LTZ, LTZ-loaded PEG-protamine nanoparticles, and empty PEG-protamine compound was formed by cross-linker TPP. Strong characteristic peaks were observed for letrozole at different peaks at 2*θ* = 11.2°, 13.12°, 14.16°, 16.24°, 17.16°, 19.72°, 21°, 21.44°, 22°, 23.16°, 25.08°, 25.52°, and 26.8°. The result regulates the crystal-like characteristics of the LTZ. The expression of the crests and alteration of the crest strength establish that the crystal-like nature of the drug is swapped to the amorphous state. The alteration and difference in crests establish the interactivity of letrozole with the PEG-protamine complex and sodium tripolyphosphate which act as a foremost driving force for the letrozole integration in the PPC. Studies on X-ray diffraction have shown that the PEG-protamine complex and the drug letrozole have peaks typical for the drug and the PEG-protamine complex (Figures 2 and 3). The variation in LTZ and PPC peaks indicates the chemical mismatch between letrozole and PPC. The decrease in concentration and the change in peak displacement determine the interaction. From the data analysis, we came to the conclusion that the characteristic peaks of LTZ and PEG-protamine complex are conjugated and have the characteristics of the amorphous nature. The outcome suggests that LTZ is present all over the nanostructure, which clarifies and meets the requirement for a satisfactory drug delivery system [21]. The pure LTZ exhibits a strong characteristic peak at 2*θ* = 11.2°, 13.12°, 14.16°, 16.24°, 17.16°, 19.72°, 21°, 21.44°, 22°, 23.16°, 25.08°, 25.52°, and 26.8°, showing its crystalline shape while PPC-NP displays amorphous structure. PPC-LTZ-NP has various crests, and peak variation indicates the crystalline type of PPC-NP loaded with LTZ.

### 3.3. Zeta Size and Zeta Potential

The ideal particle size for formulations ranged from 258 to 388 nm, confirming that the particles were nanosized; similarly, the zeta potential of F1-F4 was observed from 7.4 to 11.2 mV. Interestingly, the recorded polydispersity index of all formulations was less than 0.5 which is clearly indicated in Table 1. From the given data, it is evident that expanding the amount of the drug will increase the size of the particles, while the freezing impact could avoid the nanoparticles from agglomerating effectively, so the samples become fluffy. The ideal particle size is small, and the particle size distribution is constricted with reference to the air-drying method. These results validate that the particle size is significantly reduced during the freeze-drying process than the air-drying process.

### 3.4. Scanning Electron Microscopy (SEM) Analysis

**Figure 4:**
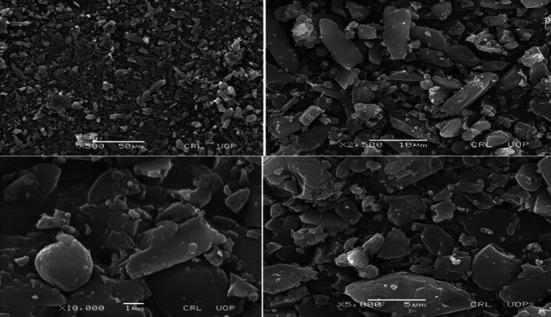
Scanning electron microscope (SEM) analysis of nanoparticle formulations F1-F4.

Surface morphology of formulated nanoparticles was determined using scanning electron microscopy (SEM). SEM images of the prepared nanoparticles showed irregularities in the shape and size, which is evident from Figure 4, where the surface is flat, free from gaps and coarseness, while the edges are intense giving a glassy appearance.

### 3.5. In Vitro Drug Release

**Figure 5:**
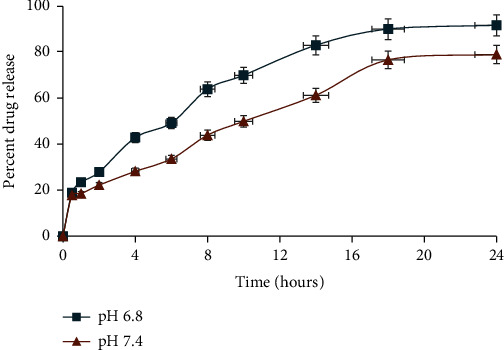
In vitro release study of letrozole from PEG-protamine complex nanoparticles at different pH (6.8 and 7.4).

A dissolution apparatus was used for dissolution studies, and data were interpreted by utilizing several kinetic models involving zero order. The kinetic model data suggested that the release of the drug followed the first-order release kinetics. The value of *R*^2^ was larger than 0.5 which showed that the drug release observed first-order kinetics and drug discharge was reliant on the quantity of the drug, which means that if the drug quantity in the polymeric matrix is high, then a large amount of drug will be released from nanoparticle complex. Moreover, the Higuchi model and Korsmeyer papers indicated that drug release from the polymeric nanoparticles is based on diffusion. During our study, drug release was noted at different pH ranging from 6.8 to 7.4; specifically, 70.2-83.9% of drug release was recorded at pH 6.8. Similarly, after 72 h dissolution, the maximum percent release at pH 6.4 and 7.4 was 89.93% and 81.95%, respectively (Figure 5) [18].

### 3.6. In Vitro Cytotoxicity Study

**Figure 6:**
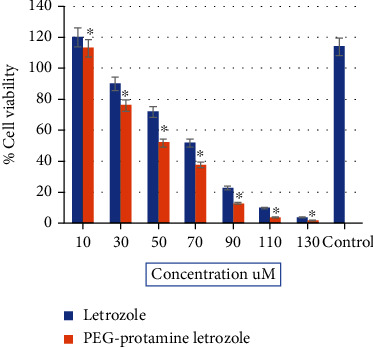
In vitro cytotoxicity assay of letrozole and PEG-protamine letrozole nanoparticle on human breast cells MCF-7.

The cytotoxicity of PEG-protamine letrozole nanoparticles were tested against the MCF-7 cells. The cells were subjected to treatment for at least 24 h, with varying quantities of letrozole and PEG-protamine letrozole NPs (10-140 *μ*M). After measuring the cell capability by the MTT assay, it was observed that both the letrozole and PEG-protamine letrozole NPs inhibited MCF-7 cellular viability in a dose-dependent manner.  Figure 6 shows the cytotoxic effect after 24 h; cell capability was assessed utilizing various amounts of letrozole and PEG-protamine letrozole NPs (10-140 *μ*M). The substantial cytotoxic effects were shown by one-way ANOVA at 50, 70, and 90 *μ*M at *p* < 0.0001 with a cell sustainability inhibition of 50%, 40%, and 20% separately as related to the control. The measured value for IC_50_ was 70 *μ*M for letrozole and 50 *μ*M for PEG-protamine letrozole NPs, which clearly indicates the effectiveness PEG-protamine letrozole nanoparticles.

Current studies on the PEG-protamine letrozole formulation have revealed a significant IC_50_ in MCF-7 cells. Future research work will be carried out to explore the mechanism leading to increased formulation efficacy and detailed in vivo study, such as typical live/dead cell staining, cellular uptake, and histological examination.

## 4. Conclusion

Letrozole has been prepared as a nanoformulation for which the method ionic gelation was adopted to integrate drug into the PEG-protamine complex with the help of a TPP connecting agent. The letrozole-loaded PEG-protamine nanoparticles exhibited a potent cytotoxic effect against MCF-7 cell lines. The calculated value for IC_50_ was 70 *μ*M for letrozole and 50 *μ*M for PEG-protamine letrozole NPs.

## Figures and Tables

**Table 1:** Particle size and potential of formulations (F1-F4).

Formulation code	PPC	LTZ	TPP	Polydispersity index	Particle size (nm)	Zeta potential
F1	30 mg	5 mg	0.4%	0.114	258	7.4 mV
F2	30 mg	10 mg	0.4%	0.234	276	8.11 mV
F3	30 mg	15 mg	0.4%	0.45	340	10.2 mV
F4	30 mg	20 mg	0.4%	0.5	388	11.2mv

## Data Availability

Upon request, data could be provided by the shared corresponding author Dr. Muhammad Imran Amirzada (imranamirzada@cuiatd.edu.pk).
